# The Chloroplast Genome of Wild *Saposhnikovia divaricata*: Genomic Features, Comparative Analysis, and Phylogenetic Relationships

**DOI:** 10.3390/genes13050931

**Published:** 2022-05-23

**Authors:** Shanyong Yi, Haibo Lu, Wei Wang, Guanglin Wang, Tao Xu, Mingzhi Li, Fangli Gu, Cunwu Chen, Bangxing Han, Dong Liu

**Affiliations:** 1 Department of Biological and Pharmaceutical Engineering, West Anhui University, Lu’an 237012, China; ysy345283991@163.com (S.Y.); weiwangwestau@163.com (W.W.); lgw93@126.com (G.W.); xutaojy@126.com (T.X.); gfl0127@163.com (F.G.); cunwuchen@163.com (C.C.); 2 Anhui Engineering Laboratory for Conservation and Sustainable Utilization of Traditional Chinese Medicine Resources, West Anhui University, Lu’an 237012, China; luhb19981005@163.com (H.L.); 02000092@wxc.edu.cn (B.H.); 3 Analytical and Testing Center, West Anhui University, Lu’an 237012, China; 4 Biodata Biotechnology Co., Ltd., Guangzhou 510555, China; limzhi87@163.com

**Keywords:** traditional Chinese medicine, complete cpDNA sequence, phylogeny

## Abstract

*Saposhnikovia divaricata*, a well-known Chinese medicinal herb, is the sole species under the genus *Saposhnikovia* of the Apiaceae subfamily Apioideae Drude. However, information regarding its genetic diversity and evolution is still limited. In this study, the first complete chloroplast genome (cpDNA) of wild *S. divaricata* was generated using de novo sequencing technology. Similar to the characteristics of *Ledebouriella seseloides*, the 147,834 bp-long *S. divaricata* cpDNA contained a large single copy, a small single copy, and two inverted repeat regions. A total of 85 protein-coding, 8 ribosomal RNA, and 36 transfer RNA genes were identified. Compared with five other species, the non-coding regions in the *S. divaricata* cpDNA exhibited greater variation than the coding regions. Several repeat sequences were also discovered, namely, 33 forward, 14 reverse, 3 complement, and 49 microsatellite repeats. Furthermore, phylogenetic analysis using 47 cpDNA sequences of Apioideae members revealed that *L. seseloides* and *S. divaricata* clustered together with a 100% bootstrap value, thereby supporting the validity of renaming *L. seseloides* to *S. divaricata* at the genomic level. Notably, *S. divaricata* was most closely related to *Libanotis buchtormensis*, which contradicts previous reports. Therefore, these findings provide a valuable foundation for future studies on the genetic diversity and evolution of *S. divaricata*.

## 1. Introduction

*Saposhnikovia divaricata* (Turcz.) Schischk., the sole species of the genus *Saposhnikovia* Schischk, under the Apiaceae subfamily Apioideae Drude, is widely distributed in the Northern regions of China. It is one of the most important and well-known traditional Chinese medicinal plants listed in the Chinese Pharmacopoeia, as well as in several pharmaceutical records, such as the Thousand Golden Prescriptions (Qian Jin Fang) and Shen Nong’s Materia Medica (Shen Nong Ben Cao Jing). The dried roots are called Fang-Feng in China, Bang-Poong in Korea, and Bofu in Japan, and have been extensively used for treating arthralgia, headaches, rheumatism, stroke, fever, and allergic rhinitis [1]. Recently, studies investigating the chemical constituents of *S. divaricata* revealed that the main active components were chromones, coumarins, and volatile oils [2,3,4], which exhibited anti-proliferative and anti-oxidant, anti-bacterial and anti-tumor, anti-convulsant, anti-coagulant, anti-inflammatory, and anti-pyretic properties [4,5,6,7,8]. However, little information is known regarding genetic diversity and evolution.

The chloroplast is a photosynthetic organelle in algae and plants that provides the energy essential for growth and reproduction by promoting the biosynthesis and metabolism of starch and fatty acids [9]. Recent studies have shown that this double membrane plant organelle originated from the endosymbiosis of cyanobacteria [10]. Chloroplast genomes (cpDNAs) are maternally inherited in most plants, and the majority of angiosperm cpDNAs are characterized by small molecules, high copy number genes, and highly conserved sequences [11,12]. Typically, cpDNAs are closed circular double-stranded DNA with a classic quadripartite structure composed of two inverted repeat regions (IRa and IRb), a small single copy (SSC) region, and a large single copy (LSC) region [13]. The cpDNA composition and sequence in angiosperms consist of highly conserved protein-coding genes (*PCGs*), transfer RNA (*tRNA*) genes, and ribosomal RNA (*rRNA*) genes [14]. However, the size, structure, and IR contraction and expansion of angiosperm cpDNAs have undergone several alterations caused by the adaptation to changing environments, and pseudocolonization even occurred in some genera [15]. Therefore, cpDNAs can be used to analyze the genetic structure and molecular characteristics among closely related plant species. The sequenced cpDNAs are generally available to download in public databases, such as the National Center for Biotechnology Information (NCBI; https://www.ncbi.nlm.nih.gov/; accessed on 14 September 2020).

With the rapid development of next-generation sequencing technology, the cpDNAs of numerous species have been fully sequenced and functionally characterized. However, although the cpDNA sequences of the cultivated *S. divaricata* in China [16,17] and its synonymous *Ledebouriella* *seseloides* from South Korea [18] have been reported, little is known about the cpDNA information of wild *S. divaricata,* especially its genetic diversity and evolutionary relationship with *L. seseloides* and other related species. *L. seseloides* has already been renamed *S. divaricata* [19], however, it is still used by some researchers [18]. Furthermore, the cpDNA sequences of *L. seseloides* and *S. divaricata* were separately published and have not yet been analyzed in one study. The genomic analysis of available data for these species will further support the validity of this renaming at the molecular level. To investigate the genetic characteristics and phylogeny of wild *S. divaricata* and to discover a molecular basis for the renaming of *L. seseloides*, we collected wild *S. divaricata* samples (33°72′ N, 112°02′ E) for high-throughput cpDNA sequencing and conducted an in-depth analysis via comparison with *L. seseloides* and other related species. Specifically, we aimed to determine the genomic features of the wild *S. divaricata* cpDNA to extensively compare its cpDNA with *L. seseloides* and other Apioideae subfamily members and to identify the repeats and simple sequence repeats (SSRs), thereby discovering the unique characteristics of the wild *S. divaricata* cpDNA. The comparison between the cpDNA sequences of wild *S. divaricata* and 46 other taxa under the subfamily Apioideae revealed their phylogenetic relationships. The repeats identified in this study may be useful for developing SSR markers to analyze the genetic diversity of Apioideae subfamily members and are candidates for DNA barcoding studies. Our findings may provide a foundation for future genomic research on the genetic diversity and evolution of *S. divaricata* and other related species.

## 2. Results and Discussion

### 2.1. Genomic Features of the Wild S. divaricata CpDNA

**Figure 1:**
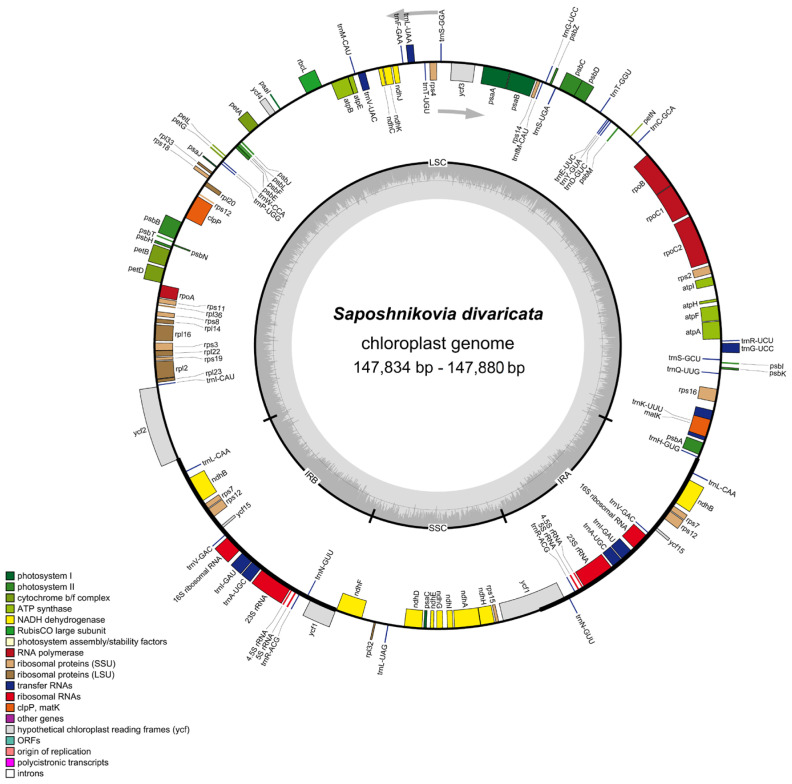
Chloroplast genome map showing all reported genes of *Saposhnikovia* *divaricata*. Genes drawn inside the circle are transcribed clockwise, and those outside are counterclockwise. Genes belonging to different functional groups are color-coded. The darker gray in the inner circle corresponds to GC content. Small single-copy (SSC) region, large single-copy (LSC) region, and inverted repeats (IRa and IRb) are displayed.

**Figure 2:**
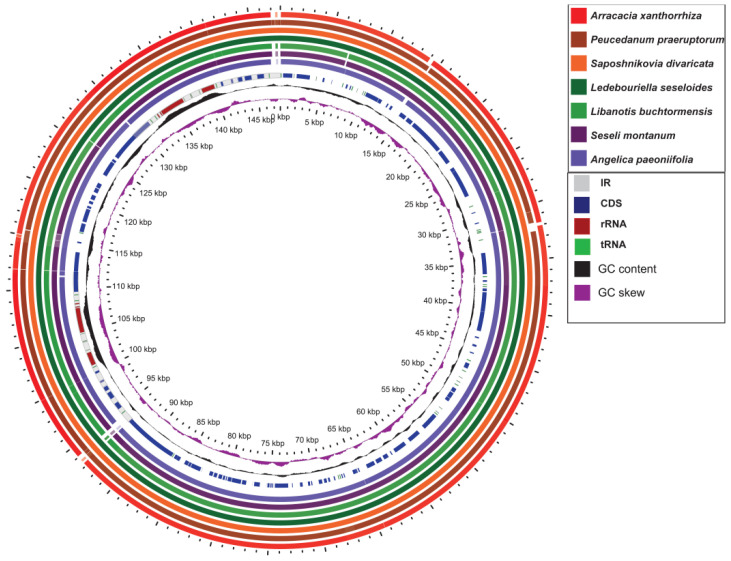
Comparison of GC content of the wild *S. divaricata* cpDNA using GView program.

The 147,834 bp-long wild *S. divaricata* cpDNA was composed of a 93,202 bp-long LSC, a 17,324 bp-long SSC, and a pair of 18,654 bp-long IR (Figure 1) regions. The IRa and IRb regions contained genes of the same type but were arranged in reverse. The length of the SSC region in wild *S. divaricata* cpDNA was similar to those of other herbs (17,000–19,000 bp), but the lengths of the IR regions were significantly shorter than those of other herbs [20,21]. The GC content of the wild *S. divaricata* cpDNA was 37.5%, which is consistent with previous reports [16,17]. Determining the GC content in the four regions is necessary for exploring species evolution and genetic relationships and is considered an important parameter for evaluating the codon preference and evolutionary trend in plants. Similar to other closely related species, the GC content in the IR regions of wild *S. divaricata* cpDNA was 44.6%, which is higher than those in the LSC (35.9%) and SSC (36.0%) regions (Table S1, Figure 2). In addition, we re-annotated, analyzed, and compared all the reported cpDNA sequences of *S. divaricata* and its synonymous species, *L. seseloides*. The results showed that the main difference between the two was the cpDNA size, although the total number of genes and unigenes was the same (Table S2).

The comprehensive and in-depth analysis of wild *S. divaricata* cpDNA revealed 129 functional genes, including 8 *rRNA* genes, 36 *tRNA* genes, and 85 *PCGs* (Table 1). Except for the double-copy gene *rps12* located in the LSC and IR regions, all genes, including eight tRNA genes (*trnA-UGC*, *trnG-UCC*, *trnI-GAU*, *trnL-CAA*, *trnL-UAA*, *trnN-GUU*, *trnR-ACG*, and *trnV-GAC*), five PCGs (*rps7*, *rps12, ndhB*, *ycf1*, and *ycf15*), and four rRNA genes (*rrn4.5*, *rrn5*, *rrn16*, and *rrn23*) were duplicated in the IR regions. Additionally, the LSC region contained 24 *tRNA* genes and 66 *PCGs*. By contrast, the SSC region only possessed one *tRNA* and twelve *PCGs*. Notably, five types of *ycf* (*ycf1*, *ycf2*, *ycf3*, *ycf4*, and *ycf15*) were detected in this genome. Moreover, two genes *(clpP* and *ycf3*) contained two introns (*rps12* was special with two copies, and its first exon was shared in the LSC region, and exons 2 and 3 were in the IR region), whereas six tRNAs (*trnK-UUU*, *trnI-GAU*, *trnA-UGC*, *trnG-UCC*, *trnV-UAC*, and *trnL-UAA*) and nine PCGs (*rps16*, *rpoC1*, *rpl2*, *rpl16*, *ndhA*, *ndhB*, *PetB*, *PetD*, and *atpF*) only possessed one intron (Table 2). The *trnK-UUU* gene had the largest intron (2532 bp), which included the *matK* gene. Introns can regulate the gene transcription rate and play a vital role in gene structure and function [22]. *Rpl2*, which was the only gene with an intron in the ribosomal large subunit of *S. divaricata* cpDNA, is commonly used as a phylogenetic marker for special species, such as those under tribe Desmodieae [23]. Screening via hybridization demonstrated that the *rpl2* intron was lost in at least five other dicotyledon lineages [24]. In higher plants, *infA* encodes approximately 70 amino acids of the translation initiation factor IF1, which is an important component of protein translation initiation in the organelles [25]. *InfA* is an extremely active gene during cpDNA evolution and has become repeatedly invalidated in 24 angiosperm lineages, although most angiosperm species seemingly contain the intact gene [26]. Furthermore, *infA* is considered the most mobile cpDNA gene in plants that has been transferred many times through evolution [27].

**Figure 3:**
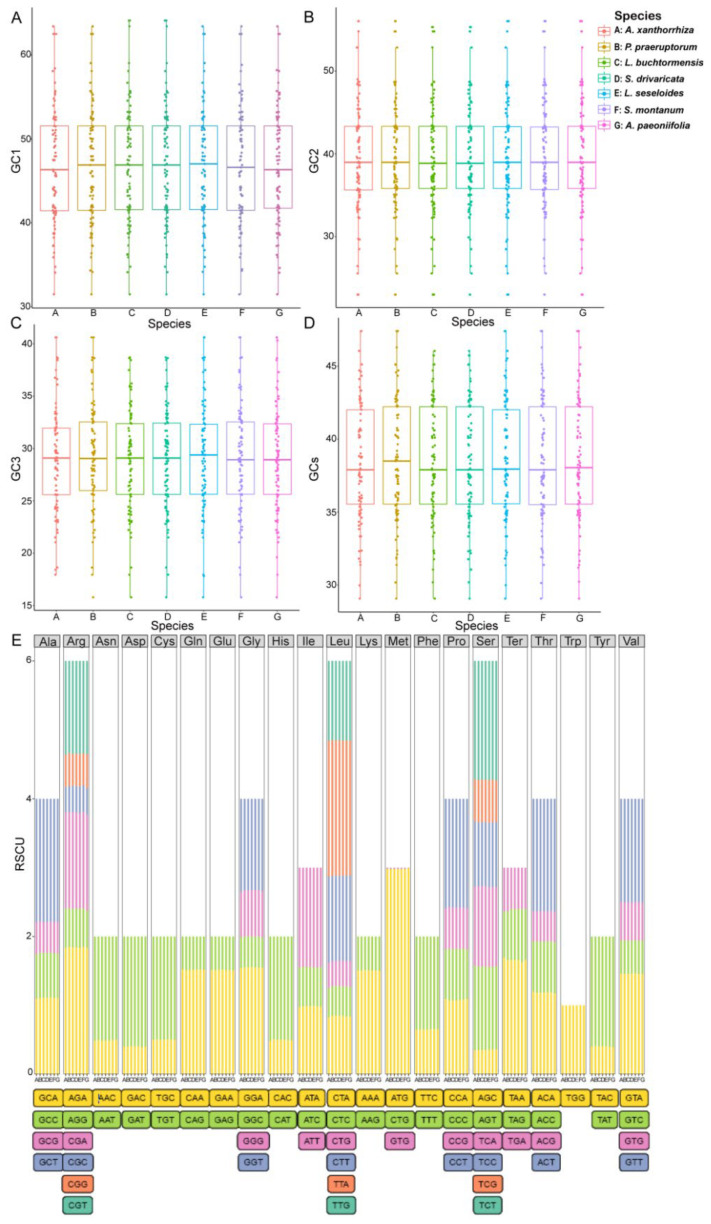
Comparison of the GC content, codon usage preference, and amino acid proportion in the protein-coding genes of seven chloroplast genomes. (**A**–**D**) GC content in the synonymous codons at the first (GC1), second (GC2), and third (GC3) positions and total GC content (GCs). (**E**) Codon preference and proportion of amino acids based on relative synonymous codon usage (RSCU) values. Ter represents the stop codon. Legend: A, *A. xanthorrhiza*; B, *P. praeruptorum*; C, *L. buchtormensis*; D, *S. divaricata*; E, *L. seseloides*; F, *S. montanum*; G, *A. paeoniifolia*.

In addition, we analyzed the codon usage preference and relative synonymous codon usage (RSCU) in the cpDNAs of *S. divaricata* and its related species. Based on the tRNA and PCG sequences, the codon usage frequency in the wild *S. divaricata* cpDNA was determined (Table S2) and compared to six closely related species, namely, *L. seseloides*, *Libanotis* *buchtormensis*, *Seseli montanum*, *Peucedanum praeruptorum*, *Angelica paeoniifolia,* and *Arracacia xanthorrhiza* (Figure 3). In total, 24,347 codons were detected in all the coding sequences of *S. divaricata*. Among these, leucine (Leu) was the most common amino acid, accounting for 10.6% (2573) of the total codons, whereas cysteine (Cys) was the least common (1.0%, 255). The comparison of the GC content in the first to third (GC1–GC3) positions and total GC content (GCs) among the seven cpDNAs indicated that the GC composition of the codons in *S. divaricata* and *L. seseloides* cpDNAs was the most similar (Figure 3A–D). Furthermore, the majority of the synonymous codons with RSCU values >1 ended with either adenine (A) or thymine (T) bases (except for TTG and ATG), indicating that codons with A or T ends are common (Table S2, Figure 3E). Notably, Arginine (Arg), Leu, and Serine (Ser) showed a high degree of codon bias among the seven species, whereas tryptophan (Trp) had no codon bias. In addition, we found that the cpDNAs of the wild *S. divaricata* and the other species preferred TAA as the termination codon. Hypothetically, the best combination of codons can promote the faster and more accurate translation of required proteins. The use of synonymous codons is also influenced by multiple factors, such as genome size, gene length, gene expression level, protein secondary structure, and gene density [28,29]. Therefore, codon preference analysis may be used to examine the balance between mutation preference and natural selection during translation optimization [30].

### 2.2. Comparative CpDNA Analysis of Seven Species under Subfamily Apioideae

**Figure 4:**
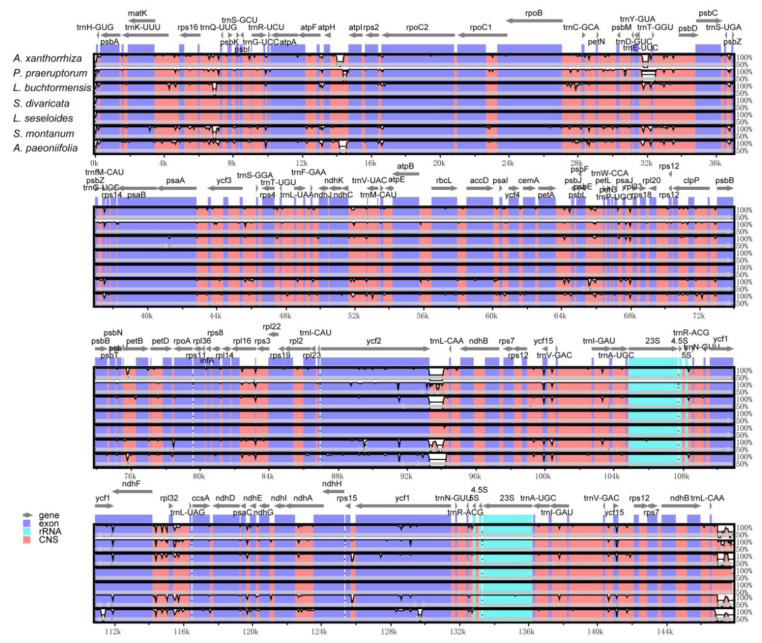
Comparison of the seven chloroplast genomes belonging to subfamily Apioideae Drude using mVISTA program. Grey arrows and thick black lines above the alignments indicate gene orientations and IR positions, respectively. A cut-off of 70% identity was used for the plots, with the Y-scale representing the percent identity (50–100%). Genome regions are color-coded as protein-coding (exon; blue), ribosomal RNA (rRNA; cyan), and conserved non-coding sequences (CNS; pink).

**Figure 5:**
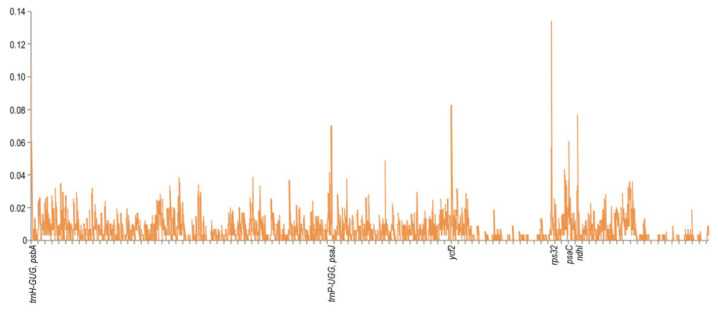
Comparison of the nucleotide variability (Pi) values among the seven species cp genomes. The Y-axis shows the Pi values; the X-axis shows the genes with high Pi values.

The sequence divergence of the cpDNAs among selected species belonging to subfamily Apioideae Drude—*L. buchtormensis* and *S. montanum* under tribe Ammineae, *P. praeruptorum* and *A. paeoniifolia* under tribe Peucedaneae, *A. xanthorrhiza* under tribe Selineae, and *L. seseloides* under tribe Laserpiteae—were examined using the *S. divaricata* (tribe Laserpiteae) cpDNA as reference (Figure 4). As expected, all cpDNAs exhibited the general structure and order of characteristic genes, with the non-coding regions showing greater variation than the coding regions. Notably, *ycf1* (IR and SSC regions) and *ycf2* (IR and LSC regions) were quite mutable. Since the lengths of *ycf1* and *ycf2* located at the boundaries of IR regions are very long, these genes are thus prone to insertion–deletion (InDel), resulting in the considerable differences between the cpDNAs of *S. divaricata* and the other species. These results indicate that the IR, SSC, and LSC regions rapidly evolved in Apioideae Drude species. Notably, the *rRNA* sequences were the most conserved among the seven *cpDNAs*, which is similar to most angiosperms, such as *Salvia miltiorrhiza* [31] and *Phyllostachys sulphurea* [32]. We also found that the degree of variation among the IR regions of the seven *cpDNAs* was low, whereas most of the variation occurred in the SSC regions and in the binding sites of the IR and LSC regions (Figure 2). In addition, all the coding regions in the seven *cpDNAs* were extracted and evaluated for nucleotide variability. Eight *PCGS*, namely, *rpl32*, *trnH-GUG*, *ycf2*, *ndhI*, *trnP-UGG*, *psaJ*, *psbA*, and *psaC*, possessed the highest Pi values, of which *rpl32* was the most variable (Figure 5).

**Figure 6:**
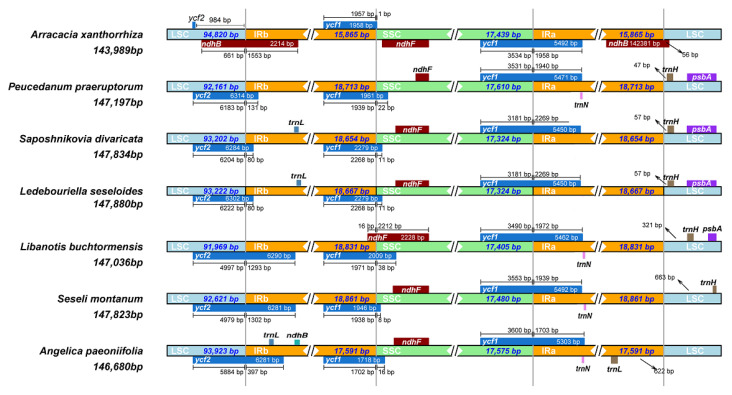
Comparison of the borders of LSC, SSC, and IR regions among seven cp genomes.

The expansion and contraction at the IR region borders are prevalent in many species and are considered the primary reason for the size differences between plant cpDNAs during evolution [33]. Comparison of the IR/LSC and IR/SSC boundaries in *A. xanthorrhiza*, *P. praeruptorum*, *S. divaricata*, *L. seseloides*, *L. buchtormensis*, *S. montanum*, and *A. paeoniifolia* was performed to assess the degree of IR expansion or contraction among these species. As expected, *S. divaricata* and *L. seseloides* contained similar boundaries in the LSC, SSC, and IR regions, with a small difference in the size of *ycf2*. This result supports the hypothesis that *S. divaricata* and *L. seseloides* are the same species. By contrast, due to the less frequent expansion of *ycf2* in the LSC/IRb junction, the IR regions in *S. divaricata* were much smaller than those in *L. buchtormensis* and *S. montanum.* In particular, the *ycf2* in the LSC region of *S. divaricata* showed an 80-bp-long expansion towards the IRb region, whereas those of *L. buchtormensis* and *S. montanum* had 1293- and 1302-bp-long expansions towards their IRb regions, respectively. The *ndhB*/*ycf2*, *ycf1*, *ndhF,* and *trnH* genes were also found to be located in the LSC/IRb, SSC/IRb, IRa/SSC, and LSC/IRa junctions, respectively (Figure 6). Among these, *ycf1*, a possible pseudogene located in the IR/SSC boundary, was generated after expansion, which is similar to the corresponding coding gene and can be considered as a non-functional genomic DNA copy. However, *ycf1* is not transcribed and has no specific physiological function. The *ycf1* sequence exhibited a 1-, 22-, 11-, 11-, 38-, 8-, and 16-bp-long expansion from the IRb to the SSC regions in the cpDNAs of *A. xanthorrhiza*, *P. praeruptorum*, *S. divaricata*, *L. seseloides, L. buchtormensis*, *S. montanum*, and *A. paeoniifolia*, respectively. By contrast, the gaps of *trnH* sequences in the LSC from the IRa regions of *P. praeruptorum*, *S. divaricata*, *L. seseloides, L. buchtormensis*, and *S. montanum* were 47, 57, 57, 321, and 663 bp long, respectively. However, *A. xanthorrhiza* and *A. paeoniifolia* contained no *trnH* in the LSC region. Notably, the majority of the cpDNAs contained *trnN* in the IRa regions, except *S. divaricata*, *L. seseloides*, and *A. xanthorrhiza*, whereas only *S. divaricata*, *L. seseloides*, and *A. paeoniifolia* possessed *trnL* in the IR regions. Moreover, *P. praeruptorum*, *L. buchtormensis*, *S. divaricata*, and *L. seseloides* possessed *psbA* in the LSC regions. Recently, the *psbA-trnH* intergenic spacer (IGS) region was used as a candidate DNA barcode sequence to identify similar species under the genus *Dendrobium* [34] and family Umbelliferae [35]. The *psbA-trnH* IGS can also be used as a barcode to distinguish whether two species belong to the same family [36]. In addition, the *trnN* in the *S. divaricata* and *L. seseloides* cpDNAs may have been lost during recombination. Therefore, we hypothesize that the *psbA-trnH* IGS can be combined with *trnN* to develop a DNA barcode for the molecular identification of *S. divaricata* plants.

### 2.3. Identification of Repeat Sequences and SSRs in Wild S. divaricata CpDNA

A total of 33 forward, 14 reverse, and 3 complement repeat sequences were discovered in the wild *S. divaricata* cpDNA (Table 3). Most of these repeats were between 20 and 50 bp in length. The largest was the 84 bp-long forward repeat in the *ycf2* of the LSC region. Notably, LSC was the region with the densest number of repeated sequences. Among these, No. 28–35 were also associated with *ycf2*, whereas No. 45 was related to *ndhA*. Ten forward repeats were located in the IR regions, including two repeats (No. 40 and 49) related to *ycf15*. Moreover, two pairs of repeats (No. 9 and 10) were found to be located in two different regions, specifically in the introns of LSC/SSC and LSC/IRb, respectively.

SSRs or microsatellites are 1–6 bp repeat sequences commonly distributed throughout the genome. SSRs have been widely employed in studies for species identification, population genetics, and evolutionary history due to their high level of intraspecific polymorphism and uniparental inheritance [37,38]. In total, forty-nine SSRs were discovered in the wild *S. divaricata* cpDNA, including forty mononucleotide (81.6%), four dinucleotide (8.2%), two trinucleotide (4.1%), and three complex (6.1%) SSRs, most of which were found in the LSC region (Table 4). Furthermore, the three complex SSRs consisted of three mononucleotide and four dinucleotide repeats. A total of 21 SSRs were detected in the genes, and the rest were located in the IGS region. Thirty-three (67.3%) mononucleotide SSRs were mainly composed of short poly A or poly T repeats and rarely contained tandem guanine (G) or cytosine (C) repeats, which corroborate previous reports on other herbs [39]. These SSR markers can be utilized for the conservation study, linkage map construction, and marker-assisted selection of wild *S. divaricata* and other closely related species.

### 2.4. Phylogenetic Analysis of 47 Taxa under Subfamily Apioideae Based on CpDNA Sequences

**Figure 7:**
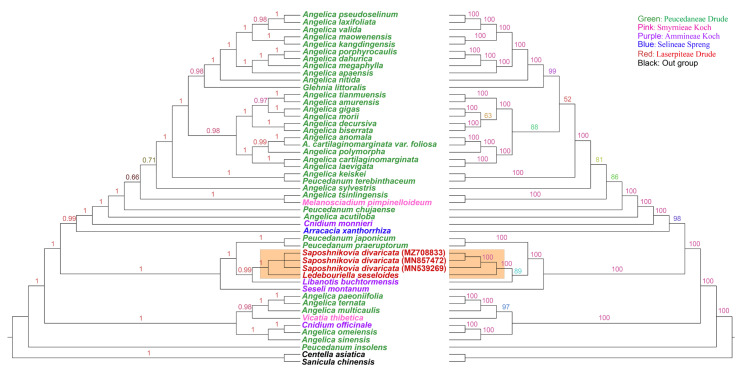
Phylogeny of 47 taxa within Apioideae Drude species based on the ML analysis of the cp genome’s IRs, LSC, and SSC regions with *Sanicula chinensis* and *Centella asiatica* as the outgroups based on FastTree (left) and IQ-TREE (right). The information of all chloroplast genomes used for phylogenetic analysis was shown in Table S3.

Based on the successful application of cpDNAs in studying angiosperm phylogeny, complete cpDNA sequences have been widely used to obtain powerful data for developing biosystem models [14]. To study the phylogenetic position of the wild *S. divaricata* within the Apiaceae subfamily Apioideae Drude, the complete cpDNAs of forty-seven taxa belonging to ten genera under tribes Peucedaneae Drude, Smyrnieae Koch, Ammineae Koch, Laserpiteae Drude, and Selineae Spreng were used for phylogenetic tree construction (Table S3). One species each from tribes Saniculoideae Drude (*Sanicula chinensis*) and Mackinlayoideae Plunkett and Lowry (*Centella asiatica*) were selected as outgroups (Figure 7). The maximum likelihood (ML) trees generated using FastTree and IQ-TREE software demonstrated similar results and ensured the reliability of the phylogenetic analysis, but also showed some difference from previous reports [16,17]. Notably, the 100% bootstrap value observed in the clustering of *L. seseloides* and *S. divaricata* further supported the hypothesis that the two were the same species. In addition, *S. divaricata* (Laserpiteae Drude) was discovered to be most closely related to *L. buchtormensis* from Ammineae Koch, *P.* *japonicum* and *P. praeruptorum* from Peucedaneae Drude, and *S. montanum* from Ammineae Koch. These results suggest that the genetic relationships between the species under genera *Saposhnikovia* and *Libanotis* are closer than those under genera *Peucedanum* and *Seseli*, as evidenced by the high bootstrap support values. Furthermore, Laserpiteae Drude and Ammineae Koch species potentially have a closer kinship with each other than with Peucedaneae Drude species, which contradicts the previous reports on cultivated *S. divaricata* [16,17].

## 3. Materials and Methods

### 3.1. Sampling, CpDNA Extraction, and Sequencing

Fresh mature leaves were plucked from wild *S. divaricata*. Total genomic DNA was extracted from young leaves using a Trelief TM Plant Genomic DNA Kit (TsingKe Biotechnology Co., Ltd., Beijing, China). After quality testing, DNA was fragmented and used to set up 350 bp short-insert libraries and the qualified libraries were sequenced with PE 150 bp on the BGISEQ-500 sequencer according to the manufacturer’s instructions. The sequencing depth was 6.0 Gb of 150-bp paired-end reads.

### 3.2. CpDNA Assembly and Annotation

First, all raw reads were trimmed using Fastp [40]. Subsequently, high-quality reads were mapped to the reference chloroplast genomes of Apioideae obtained from GenBank through Bowtie2 v.2.3.4.3 (Langmead B, et al. https://github.com/BenLangmead/bowtie2, accessed on accessed on 14 September 2020) [41]. The sequence of the coding gene having the maximum coverage was utilized as a seed sequence for de novo assembly by NOVOPlasty v4.2.1 [42]. The assembled cp genomes were annotated with DOGMA [43], GeSeq [44], tRNAscan [45], and ARAGORN [46], then manually adjusted and confirmed using Geneious 9.1.8 (M Kearse, et al. San Diego, CA, https://www.geneious.com/, accessed on accessed on 14 September 2020) [47]. The circular chloroplast genome map was drawn by OrganellarGenomeDRAW tool (OGDRAW) v.1.3.1 (Greiner S, et al. https://chlorobox.mpimp-golm.mpg.de/OGDraw.html, accessed on accessed on 14 September 2020) [48] for further comparison of gene order and content. The other genomes downloaded from GenBank for comparative analysis were re-annotated according to the above method. The assembled cp genome has been deposited to the GenBank with the accession number MZ708833.

### 3.3. CpDNA Comparison and Sequence Divergence Analysis

The Relative Synonymous Codon Usage (RSCU) values were determined to quantify the extent of the codon usage bias. RSCU was calculated for every codon in each genome according to the published equation [49]. The overall GC content and GC content at the first, second, and third codon positions (GC1, GC2, and GC3, respectively) of the genomes were calculated using EMBOSS software suite [50]. Simple sequence repeats (SSRs) were searched via MISA v1.01 [51] with the following criteria: 10, 6, 5, 5, 5, and 5 repeat units for mono-, di-, tri-, tetra-, penta-, and hexa-nucleotides, respectively. Chloroplast genome similarity was assessed using BLAST Atlas on the GView server (Franklin B., et al. https://server.gview.ca/, accessed on 14 September 2020) [52] with *S. divaricata* genome as a reference. The junction regions between the IR, SSC, and LSC of these plastomes were compared using the IRscope+ online program [53]. The divergent regions were visualized using Shuffle-LAGAN mode [54] included in mVISTA v.2.0 (Frazer K.A., et al., https://genome.lbl.gov/vista/mvista, accessed on 14 September 2020) [55] with *S. divaricata* genome as a reference. To identify polymorphic regions with substantial variability, the aligned sequences were imported in DnaSP v6.12.03 (DNA Sequences Polymorphism) (Rozas J., et al. http://www.ub.edu/dnasp/, accessed on 14 September 2020) using the sliding window method with a step size of 15 bp and a window length of 200 bp [56].

### 3.4. Phylogenetic Analysis

The complete cp genomes of forty-seven taxa from the Apiaceae subfamily Apioideae Drude and two species from Saniculoideae Drude (*S. chinensis*) and Mackinlayoideae Plunkett and Lowry (*C. asiatica*) as outgroups were employed for the phylogenetic reconstruction. These cpDNAs were downloaded from GenBank in NCBI (Table S3). The whole cpDNA sequence alignment was carried out by using MAFFT v7.450 (Katoh K., et al. https://mafft.cbrc.jp/alignment/software/, accessed on 14 September 2020) [57], and then the regions with consistent site coverage less than 95% were deleted. Maximum likelihood (ML) analysis was performed by FastTree 2.1.11 (Price M.N., et al. http://www.microbesonline.org/fasttree/, accessed on 14 September 2020) [58] and IQ-TREE version 2.1.4 (Minh B.Q., et al. https://github.com/iqtree/iqtree2, accessed on 14 September 2020) [59]. The former was conducted under the best-fit nucleotide substitution model with General Time Reversible + γ (GTR + γ), Shimodaira–Hasegawa test, and the latter was determined using the Akaike Information Criterion (AIC) by ModelFinder in the IQ-TREE package and 1000 bootstrap replicates [60].

## 4. Conclusions

In this study, we first analyzed the cpDNA of the wild *S. divaricata* and compared it with its close relatives. The wild *S. divaricata* cpDNA contained 8 *rRNA* genes, 36 *tRNA* genes, and 85 *PCGs* and had a total GC content of 37.5%. These results are consistent with all the reported cpDNA sequences of *S. divaricata* and its synonymous species, *L. seseloides*. Compared to other related species, the non-coding regions exhibited greater variation than the coding regions. The comparison of the IR/LSC and IR/SSC boundaries among seven cpDNAs revealed that the *trnN* in the wild *S. divaricata* may have been lost during the reorganization process. Hence, *trnN* can be combined with the *psbA-trnH* IGS region as a DNA barcode for the Apioideae Drude species. We also found that the LSC region was a dense region of repeated sequences, in which 49 potentially informative SSRs were identified. Furthermore, the genetic relationship between *L. seseloides* and *S. divaricata* was confirmed at the genomic level for the first time. Notably, these two were most closely related to *L. buchtormensis,* which contradicts previous reports. By contrast, the phylogenetic tree showed that the Laserpiteae Drude and Ammineae Koch species have a close kinship. Overall, our findings contribute important genetic information that may be useful for future studies on the genetic diversity and phylogenetic relationships of the Apioideae species.

## Figures and Tables

**Table 1:** List of genes found in the chloroplast genome of the wild *S. divaricata*.

	Group of Genes	Gene Name	Number
**Self-replication**	*rRNA* genes	*rrn4.5* (×2), *rrn5* (×2), *rrn16* (×2), *rrn23* (×2)	8
	*tRNA* genes	* trnA-UGC (×2), *trnC-GCA*, *trnD-GUC*, *trnE-UUC*, *trnF-GAA*, *trnfM-CAU*, * trnG-UCC (×2), *trnH-GUG*, *trnM-CAU*, * trnI-GAU (×2), * trnK-UUU, *trnL-CAA* (×2), * trnL-UAA (×2), *trnI-CAU*, *trnN-GUU* (×2), *trnP-UGG*, *trnQ-UUG*, *trnR-ACG* (×2), *trnR-UCU*, *trnS-GCU*, *trnS-GGA*, *trnS-UGA*, trnT-GGU, *trnT-UGU*, *trnV-GAC* (×2), * trnV-UAC, *trnW-CCA*, *trnY-GUA*	36
	Ribosomal small subunit	*rps2*, *rps3*, *rps4*, *rps7* (×2), *rps8*, *rps11*, *rps12* (×2), *rps14*, *rps15*, * rps16, *rps18*, *rps19*	14
	Ribosomal large subunit	* rpl2, *rpl14*, * rpl16, *rpl20*, *rpl22*, *rpl23*, *rpl32*, *rpl33*, *rpl36*	9
	DNA-dependent RNA polymerase	*rpoA*, *rpoB*, * rpoC1, *rpoC2*	4
**photosynthesis**	Large subunit of rubisco	*rbcL*	1
	Photosystem I	*psaA*, *psaB*, *psaC*, *psaI*, *psaJ*	5
	Photosystem II	*psbA*, *psbB*, *psbC*, *psbD*, *psbE*, *psbF*, *psbH*, *psbI*, *psbJ*, *psbK*, *psbL*, *psbM*, *psbN*, *psbT*, *psbZ*	15
	NADH dehydrogenase	* ndhA, * ndhB (×2), *ndhC*, *ndhD*, *ndhE*, *ndhF*, *ndhG*, *ndhH*, *ndhI*, *ndhJ*, *ndhK*	12
	Cytochrome b/f complex	*petA*, * petB, * petD, *petG*, *petL*, *petN*	6
	ATP synthase	*atpA*, *atpB*, *atpE*, * atpF, *atpH*, *atpI*	6
**other**	Maturase	*matK*	1
	Subunit of acetyl-CoA carboxylase	*accD*	1
	Envelope membrane protein	*cemA*	1
	Protease	** clpP	1
	C-type cytochrome synthesis	*ccsA*	1
	Translation initiation factor	*infA*	1
**Functions unknown**	Conserved open reading frames	*ycf1* (×2), *ycf2*, ** ycf3, *ycf4*, *ycf15* (×2)	7
**Total**			129

One star character (*) means one intron; (**) means two introns; (×2) indicates genes with two copies.

**Table 2:** The genes with introns in the wild *S. divaricata* chloroplast genome and the length of the exons and introns.

Gene	Location	Exon1 (bp)	Intron1 (bp)	Exon2 (bp)	Intron2 (bp)	Exon3 (bp)
*trnK-UUU*	LSC	37	2532	35		
*trnI-GAU*	IRb	37	968	35		
*trnI-GAU*	IRa	37	968	35		
*t* *rnA-UGC*	IRb	38	818	35		
*t* *rnA-UGC*	IRa	38	818	35		
*trnG-UCC*	LSC	23	703	48		
*trnV-UAC*	LSC	39	569	35		
*trnL-UAA*	LSC	35	502	50		
^1^ *rps12*	LSC + IRa	114		232	538	26
^1^ *rps12*	LSC + IRb	114		232	538	26
*rps16*	LSC	40	859	197		
*rpoC1*	LSC	432	748	1605		
*rpl2*	LSC	394	651	434		
*rpl16*	LSC	9	950	399		
*ndhA*	SSC	553	1099	539		
*ndhB*	LSC	777	682	756		
*ndhB*	IRa	777	682	756		
*PetB*	LSC	6	758	642		
*PetD*	LSC	8	750	475		
*atpF*	LSC	145	711	401		
*clpP*	LSC	231	635	292	848	71
*ycf3*	LSC	153	776	228	717	126

^1^ Since the *rps12* gene is trans-spliced in the wild *S. divaricata* cpDNA, the length of intron 1 is not counted.

**Table 3:** Repeat sequences in the chloroplast genome of the wild *S. divaricata*.

ID	Size (bp)	Repeat 1	Type ^1^	Size (bp)	Repeat 2	Mismatch (bp)	E-Value	Gene	Region
1	34	7110	F	32	7126	3	0.00011	IGS	LSC
2	32	8400	F	31	36,451	2	1.90 × 10^−5^	IGS	LSC
3	34	9846	R	32	115,685	3	0.00011	IGS	LSC;SSC
4	35	9851	C	34	115,668	3	3.00 × 10^−5^	IGS	LSC;SSC
5	35	9851	C	32	115,670	3	3.00 × 10^−5^	IGS	LSC;SSC
6	35	20,788	F	35	20,837	3	3.00 × 10^−5^	IGS	LSC
7	36	32,147	R	37	32,160	3	2.23 × 10^−6^	IGS	LSC
8	39	44,679	F	39	98,962	2	1.74 × 10^−9^	*ycf3* (intron); IGS	LSC;IRb
9	39	44,679	F	39	122,483	3	1.64 × 10^−7^	*ycf3* (intron); *ndhA* (intron)	LSC;SSC
10	35	44,682	F	35	95,893	3	3.00 × 10^−5^	*ycf3* (intron); *ndhB*	LSC;IRb
11	33	44,685	F	33	98,968	1	3.27 × 10^−8^	*ycf3* (intron); IGS	LSC;IRb
12	31	51,905	R	31	64,144	1	4.92 × 10^−7^	IGS	LSC
13	35	51,907	R	35	51,907	2	3.57 × 10^−7^	IGS	LSC
14	32	51,907	F	32	51,923	2	1.90 × 10^−5^	IGS	LSC
15	37	51,911	F	36	64,143	3	2.23 × 10^−6^	IGS	LSC
16	42	51,912	R	42	51,912	2	3.15 × 10^−11^	IGS	LSC
17	42	51,912	R	40	51,912	2	3.15 × 10^−11^	IGS	LSC
18	28	51,912	F	28	115,670	0	8.53 × 10^−8^	IGS	LSC;SSC
19	31	51,912	R	31	115,663	1	4.92 × 10^−7^	IGS	LSC;SSC
20	36	51,913	R	39	51,916	3	1.64 × 10^−7^	IGS	LSC
21	35	51,914	R	37	115,674	3	2.23 × 10^−6^	IGS	LSC;SSC
22	33	51,922	F	32	115,663	2	5.07 × 10^−6^	IGS	LSC;SSC
23	30	51,925	R	29	115,669	1	1.90 × 10^−6^	IGS	LSC;SSC
24	32	52,673	R	32	52,673	2	1.90 × 10^−5^	IGS	LSC
25	31	64,142	R	31	64,142	2	7.14 × 10^−5^	IGS	LSC
26	35	64,144	C	36	115,671	3	8.18 × 10^−6^	IGS	LSC;SSC
27	25	67,922	F	25	67,946	0	5.46 × 10^−6^	IGS	LSC
28	84	91,433	F	84	91,451	1	1.64 × 10^−38^	*ycf2*	LSC
29	70	91,433	F	70	91,469	3	2.12 × 10^−25^	*ycf2*	LSC
30	52	91,433	F	52	91,487	3	5.90 × 10^−15^	*ycf2*	LSC
31	59	91,440	F	59	91,476	1	1.30 × 10^−23^	*ycf2*	LSC
32	45	91,440	F	45	91,494	2	5.66 × 10^−13^	*ycf2*	LSC
33	59	91,458	F	59	91,476	0	1.85 × 10^−26^	*ycf2*	LSC
34	41	91,458	F	41	91,494	0	1.27 × 10^−15^	*ycf2*	LSC
35	23	91,458	F	23	91,512	0	8.73 × 10^−5^	*ycf2*	LSC
36	44	94,003	F	44	94,024	1	1.04 × 10^−14^	IGS	IRb
37	36	94,011	F	36	94032	0	1.30 × 10^−12^	IGS	IRb
38	41	98,960	F	41	122,481	3	1.19 × 10^−8^	IGS;ndhA (intron)	IRb;SSC
39	33	98,968	F	33	122,489	2	5.07 × 10^−6^	IGS;ndhA (intron)	IRb;SSC
40	42	99,905	F	42	99,926	0	3.18 × 10^−16^	*ycf15*	IRb
41	34	107,943	F	34	107,975	1	8.43 × 10^−9^	IGS	IRb
42	31	108,296	F	31	132,709	2	7.14 × 10^−5^	IGS	IRb;IRa
43	23	114,349	F	23	114,381	0	0.0000873	IGS	SSC
44	28	115,668	R	28	115,668	0	8.53 × 10^−8^	IGS	SSC
45	31	122,640	R	31	122,640	0	1.33 × 10^−9^	*ndhA* (intron)	SSC
46	34	133,027	F	34	133,059	1	8.43 × 10^−9^	IGS	IRa
47	31	133,030	F	30	133,063	2	7.14 × 10^−5^	IGS	IRa
48	23	133,038	F	23	133,070	0	0.0000873	IGS	IRa
49	42	141,068	F	42	141,089	0	3.18 × 10^−16^	*ycf15*	IRa
50	44	146,968	F	44	146,989	1	1.04 × 10^−14^	IGS	IRa

^1^ F, Forword; R, Reverse, C, complement; IGS, intergenic space.

**Table 4:** Simple sequence repeats (SSRs) in the wild *S. divaricata* chloroplast genome.

ID	Type	Repeat Motif	bp	Start	End	Region	Gene	ID	Type	Repeat Motif	bp	Start	End	Region	Gene
1	p1	(A)10	10	1539	1548	LSC		26	c	(A)11gacaggtttttgctccttttcgtataatattcttgtattcttgtaa Tagaaaaataatagaaaag (A)10	86	71,813	71,898	LSC	*clpP*
2	p1	(A)10	10	1794	1803	LSC	*trnK-UUU*	27	p1	(T)10	10	72,637	72,646	LSC	
3	p3	(TTA)5	15	5419	5433	LSC	*rps16*	28	p1	(T)12	12	83,124	83,135	LSC	*rpl16*
4	p1	(A)10	10	9393	9402	LSC	*trnR-UCU*	29	p1	(T)16	16	84,843	84,858	LSC	
5	p2	(AT)7	14	9867	9880	LSC		30	p1	(G)13	13	94,287	94,299	IRb	
6	p2	(AT)9	18	13,059	13,076	LSC		31	p1	(T)13	13	99,335	99,347	IRb	
7	p1	(A)14	14	16,406	16,419	LSC		32	p1	(T)10	10	103,203	103,212	IRb	*trnI-GAU*
8	p1	(T)11	11	18,651	18,661	LSC	*rpoC2*	33	p1	(G)14	14	104,444	104,457	IRb	*trnA-UGC*
9	p1	(T)12	12	26,380	26,391	LSC		34	p1	(A)10	10	111,049	111,058	IRb	*ycf1*
10	p1	(A)10	10	27,392	27,401	LSC		35	p1	(A)11	11	111,833	111,843	IRb	*ycf1*
11	p3	(AAT)6	18	28,642	28,659	LSC		36	p1	(A)12	12	115,539	115,550	SSC	
12	p1	(T)12	12	29,602	29,613	LSC		37	c	(TA)6ttt(TA) 8aattatatatatga(AT)6	57	115,669	115,725	SSC	
13	p1	(T)12	12	32,753	32,764	LSC		38	p1	(A)13	13	116,776	116,788	SSC	*ccsA*
14	p1	(A)12	12	33,320	33,331	LSC		39	p1	(A)11	11	120,333	120,343	SSC	
15	p1	(C)10	10	37,141	37,150	LSC		40	p1	(T)10	10	121,130	121,139	SSC	
16	p1	(A)13	13	43,455	43,467	LSC		41	p1	(T)15	15	128,046	128,060	SSC	
17	p1	(T)10	10	45,269	45,278	LSC	*ycf3*	42	p1	(T)11	11	128,410	128,420	SSC	*ycf1*
18	p2	(TA)7	14	47,465	47,478	LSC		43	p1	(T)10	10	128,671	128,680	SSC	*ycf1*
19	p2	(TA)7	14	51,926	51,939	LSC		44	p1	(T)11	11	129,194	129,204	IRa	
20	p1	(T)10	10	52,688	52,697	LSC		45	p1	(T)10	10	129,979	129,988	IRa	
21	p1	(T)10	10	55,648	55,657	LSC	*atpB*	46	p1	(C)14	14	136,580	136,593	IRa	*trnA-UGC*
22	p1	(A)18	18	56,234	56,251	LSC		47	p1	(A)10	10	137,825	137,834	IRa	*trnI-GAU*
23	p1	(T)10	10	58,021	58,030	LSC		48	p1	(A)13	13	141,690	141,702	IRa	
24	p1	(T)10	10	60,531	60,540	LSC		49	p1	(C)13	13	146,738	146,750	IRa	
25	c	(A)10tatcagaacttt (TA)6	34	64,123	64,156	LSC									

## Data Availability

The data presented in this study are openly available in GeneBank.

## Supplementary Materials

The following are available online at https://www.mdpi.com/article/10.3390/genes13050931/s1, Table S1: Base composition in the chloroplast genome of the wild *S. divaricata*. Table S2: All chloroplast genome information of *S. divaricata* and its synonyms. Table S3: Codon-anticodon recognition patterns and codon usage for the wild *S. divaricata* chloroplast genome. Table S4: The information of all complete chloroplast genomes of Apioideae Drude used for phylogenetic analysis in this study.
